# The genitofemoral nerve block: a method for hemiscrotal anaesthesia at the bedside

**DOI:** 10.1308/003588412X13373405385133

**Published:** 2012-09

**Authors:** MD Stringer

**Affiliations:** Otago School of Medical Sciences, University of Otago, Dunedin,New Zealand

## Abstract

Pankhania M, Ali S. The genitofemoral nerve block: a method for hemiscrotal anaesthesia at the bedside. *Ann R Coll Surg Engl* 2012; **94**: 272. doi 10.1308/003588412X13171221591259a

The anatomical interpretation of genitofemoral nerve block reported by Pankhania and Ali is rather simplistic for two reasons:

1. Injection of local anaesthetic just lateral to the spermatic cord at the level of the pubic tubercle will almost certainly anaesthetise cutaneous branches of the ilioinguinal nerve together with the genital branch of the genitofemoral nerve. In most individuals, both nerves contribute variably to the ipsilateral sensory supply of the scrotum.^1^
2. The anatomy of the genital branch of the genitofemoral nerve is highly variable. It does not often lie ‘immediately lateral to the spermatic cord as it emerges from the superficial inguinal ring’. In most men it innervates the cremaster muscle and is therefore a content of the spermatic cord within the inguinal canal but at the superficial inguinal ring it is usually found on the dorsal aspect of the spermatic cord.^1,2^ Occasionally, it is absent or communicates directly with the ilioinguinal nerve.^1^
